# Bacteriophage Interactions with Marine Pathogenic Vibrios: Implications for Phage Therapy

**DOI:** 10.3390/antibiotics7010015

**Published:** 2018-02-24

**Authors:** Panos G. Kalatzis, Daniel Castillo, Pantelis Katharios, Mathias Middelboe

**Affiliations:** 1 Marine Biological Section, University of Copenhagen, DK-3000 Helsingør, Denmark; panos.kalatzis@bio.ku.dk (P.G.K.); daniel.castillo@bio.ku.dk (D.C.); 2 Institute of Marine Biology, Biotechnology and Aquaculture, Hellenic Centre for Marine Research, 71500 Crete, Greece; katharios@hcmr.gr

**Keywords:** marine vibrios, bacteriophages, phage therapy, biological control, aquaculture, interactions, vibriosis

## Abstract

A global distribution in marine, brackish, and freshwater ecosystems, in combination with high abundances and biomass, make vibrios key players in aquatic environments, as well as important pathogens for humans and marine animals. Incidents of *Vibrio*-associated diseases (vibriosis) in marine aquaculture are being increasingly reported on a global scale, due to the fast growth of the industry over the past few decades years. The administration of antibiotics has been the most commonly applied therapy used to control vibriosis outbreaks, giving rise to concerns about development and spreading of antibiotic-resistant bacteria in the environment. Hence, the idea of using lytic bacteriophages as therapeutic agents against bacterial diseases has been revived during the last years. Bacteriophage therapy constitutes a promising alternative not only for treatment, but also for prevention of vibriosis in aquaculture. However, several scientific and technological challenges still need further investigation before reliable, reproducible treatments with commercial potential are available for the aquaculture industry. The potential and the challenges of phage-based alternatives to antibiotic treatment of vibriosis are addressed in this review.

## 1. Vibrios in Marine Ecosystems

The Vibrionaceae family, and more specifically, the genus *Vibrio*, encompasses genetically and metabolically diverse, heterotrophic bacteria that can thrive in a great range of habitats. The particularly versatile features of vibrios have made them ubiquitous components of world’s marine and even brackish or freshwater ecosystems [1]. The relatively high abundance (often 10^3^ to 10^4^ cells per mL) and biomass of vibrios in the oceans [2], makes them important players in marine biogeochemical cycling. Key traits supporting this are (a) their ability to survive for a long time under nutrient-limited conditions [3,4], (b) their ability to maintain high ribosome content, which helps them achieve a fast recovery from starvation as soon as carbon sources become available [5,6,7], and c) their chemotactic response in finding nutrient sources [8,9,10,11].

The vast majority of vibrios occupy ecological niches associated with attachment to living organisms, which provide them protection and nutrients [12,13,14]. However, vibrios also occur as free-living cells in the water column [15,16]. Among several environmental variables that have been examined, salinity and temperature have been consistently linked to the observed variation in the total *Vibrio* abundance in the water column [17,18]. For example, *V. vulnificus* could tolerate a broad range in salinity from 5 to 38 ppt [19], while *V. cholerae* can grow in salinities of up to 45 ppt, if the nutrient concentration is high [20]. High temperature significantly boosts the growth of vibrios and the increased sea surface temperature has been suggested to promote a long-term increase in *Vibrio* abundance [21,22].

Their opportunistic lifestyle features, as well as their easy cultivation under laboratory conditions, have made them ideal models for investigations of bacterial population biology and genomics, disease dynamics, bacteria-phage interactions, and quorum sensing (QS) [23,24,25,26].

Several *Vibrio* species are pathogenic, and constitute a serious threat for human health. Over 80 species have been described, and at least 12 of them are known human pathogens [27,28]. *V. cholerae*, the causative agent of epidemic cholera, was introduced in Europe via sea trade routes from Asia, and was a devastating disease during 1817–1923 [29]. *V. cholerae*, as well as the seafood poisoning agents *V. parahaemolyticus* and *V. vulnificus* [30], have aroused significant attention among the scientific community, especially today, when increases in *Vibrio*-associated disease outbreaks in response to elevated ocean temperatures [31,32,33] emphasize the increasing importance of vibrio pathogens in a future warmer climate [34,35].

## 2. Vibrios in Aquaculture

According to Food and Agriculture Organization (FAO) [36], aquaculture is one of the most rapidly growing sectors for animal food production, supporting approximately 50% of the global human fish consumption. Vibrios have been characterized as the “scourge” of marine fish and shellfish, since several members of the genus can be the causative agents of a fatal disease, commonly known as vibriosis [37]. Sudden vibriosis outbreaks have been causing severe losses in biomass, with significant economic consequences for the aquaculture industry [38]. Furthermore, lower growth rate of sick fish and shellfish, excessive waste of fish feeds, and finally, the increased skepticism of consumers about aquaculture’s quality and credibility, are also important consequences of vibriosis.

Sustainability in aquaculture demands a thorough and sophisticated disease management plan in which the issue of pathogenic vibrios should be an integral part. The last report of the World Bank about prospects for fisheries and aquaculture [39] is a case in point, since it was reported that *Vibrio*-caused disease designated as early mortality syndrome (EMS), or else, acute hepatopancreatic necrosis disease (AHNPD), is a rapidly emerging disease, and a serious setback to the shrimp rearing industries of Asia and America [40,41]. FAO has drawn special attention to vibriosis [36], because the distribution of vibrios is being shifted according to the changing warming patterns, hence, outbreaks tend to be observed even in temperate or cold regions [42].

*V. anguillarum*, initially reported as *Bacillus anguillarum* [43], used to be the first isolated *Vibrio* to which “Red Pest of eels” was attributed, during early 1900s [44]. Although it still remains a serious threat for aquaculture [45,46], a plethora of other *Vibrio* species have been recorded in the literature as causative agents of vibriosis in aquaculture. *V. harveyi*, *V. parahaemolyticus*, *V. alginolyticus*, *V. vulnificus*, and *V. splendidus* [28,47,48,49,50,51,52] are the most important, while the list is expanding with the discovery of new pathogenic species, such as *V. owensii* [53].

**Figure 1:**
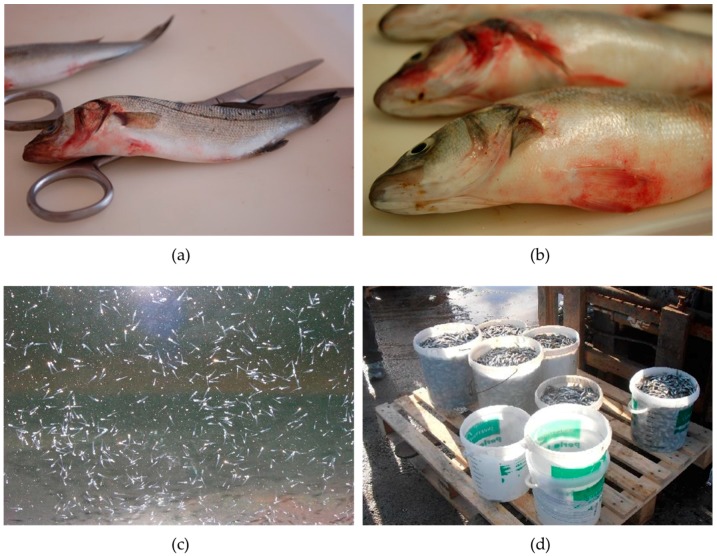
Massive mortalities caused by vibriosis in different developmental stages. (**a**,**b**) cultured European seabass, *Dicentrarchus labrax*, (**c**) cultured European seabass, *Dicentrarchus labrax* fry and (**d**) cultured gilthead sea bream, *Sparus aurata* larvae in the hatchery.

Chemical stressors, such as poor water quality and diet composition, biological stressors, such as population density and presence of other micro- or macro-organisms, and physical stressors, such as temperature above 15 °C, are the most important factors triggering vibriosis outbreaks [46,54]. Although the regulatory mechanisms of virulence in vibrios still need to be elucidated, virulence-related factors and genes that have been found in several pathogenic marine *Vibrio*. Iron uptake systems of *V. ordalii*, *V. vulnificus*, *V. alginolyticus*, and *V. anguillarum* have been recorded to contribute to their virulence, by binding the iron attached to the siderophore proteins of their hosts [46,50,55]. Extracellularly secreted proteins can have proteolytic, hydrolytic, hemolytic, and cytotoxic activity in several pathogenic *Vibrio*, such as *V. anguillarum*, *V. alginolyticus, V. harveyi, V. splendidus*, and *V. pelagius* [56,57,58,59,60]. However, the presence of virulence genes alone is not always a sufficient condition for a virulent phenotype. For instance, both virulent and avirulent *V. harveyi* and *V. campbellii* do carry virulence genes. It has been found that virulence can be coordinated via cell to cell communication, regulated by the presence of specific signal molecules [61]. The three-channel QS system of *V. campbellii*, previously described as *V. harveyi* [62], is a well-described case of QS-regulated virulence system using three different signals [63]. It was also recently shown that the virulence of *V. anguillarum* against European seabass (*Dicentrarchus labrax*) larvae is regulated by the indole signaling molecule pathway [64]. Clinical signs of vibriosis (Figure 1) include lethargic behavior, loss of appetite, unusual swimming behavior close to the water surface, increased mucus secretion, as well as petechiae and hemorrhages on their skin. Additional symptoms of the disease commonly observed are intestinal necrosis, anemia, ascetic fluid, petechial hemorrhages in the muscle wall, and liquid in the swim bladder [65,66].

A vibriosis outbreak may have catastrophic consequences for both the cultured animals and the producer, hence, implementation of preventive strategies is the safest way to cope with such potential events. Development of vaccines against vibriosis has so far been quite successful, since it has managed to significantly prevent the outbreaks of the disease [67], yet there are still important issues to be addressed. While fish are still in the hatchery, their immune system is not completely developed yet, thus vaccination is inefficient at this stage. Additionally, vaccination of juvenile fish by injection is difficult, so they are vaccinated orally and/or by immersion, resulting in low efficacy and short protection [68]. However, this is not the case for all vibrios, since immersion vaccination against *V. anguillarum* has been shown to provide a high level of protection. The biggest problem, however, is the lack of commercially available vaccines for the majority of the pathogenic *Vibrio* species other than *V. anguillarum*. Administration of antibiotics is therefore the most commonly applied strategy to tackle vibriosis outbreaks. However, if applied in marine hatcheries, antibiotics disturb the natural microbial balance in the water, as well as the developing microbiota of the larvae [69]. Furthermore, the excessive amount of antibiotics that have been used, not only for treatment, but even for prophylaxis during the last decades, has become a constantly growing problem for human and animal health, as well as for the environment [70]. There is a fundamental difficulty in controlling the amount and types of antibiotics that are applied, since the regulations for their usage can vary broadly among different countries. Development of multi-drug resistant strains, disturbance of natural microbiota, environmental residues, and public health issues, are only some of the most important problems caused by the excessive use of chemotherapy [71], and new alternatives are necessary.

## 3. Lytic Bacteriophages against Marine Vibrios

The use of bacteriophages against pathogenic bacteria in aquaculture was first introduced experimentally in Japan against *Lactococcus garvieae* in 1999 [72], and it has since been a topic of great interest for the scientific community [73,74,75,76]. Vibrios have been one of the main targets for bacteriophage isolation because of their high pathogenicity, broad presence, and ability to infect cultured fish and shellfish at various culture stages. Several potent phages have been tested against vibriosis causative agents, such as *V. harveyi*, *V. parahaemolyticus*, *V. alginolyticus*, *V. splendidus, V. anguillarum*, and *V. coralliilyticus* (Table 1), leading, in all cases, to increased survival rates of the cultured animals.

Biological treatment of *V. harveyi*-caused vibriosis has been quite successful in *Penaeus monodon* shrimp hatcheries. Vinod and colleagues [77] performed both short-term and long-term phage treatment trials using a broad host range, lytic siphovirus. During the short-term trials (48 h), the lytic vibriophage was administered as phage suspension at low multiplicity of infection (MOI = 1) to post-larval shrimps (18 days) that were previously infected by *V. harveyi*. Both single-dose (0 h) and double-dose (0 and 24 h) phage administration, led to 70% shrimp survival along with a 2-log reduction of *V. harveyi*, and 80% shrimp survival along with a 3-log reduction of *V. harveyi*, respectively. By contrast, controls without phage treatment showed only 25% survival and a 1-log increase of *V. harveyi*. During long-term trials (17 days), 35,000 naturally *V. harveyi*-infected nauplii were treated with the lytic vibriophage on a daily basis, and their average survival was 86%, compared to only 17% in the non-treated nauplii. Compared to antibiotics, which only led to a 40% survival, phage therapy provided better protection for infected shrimp. Similarly, Karunasagar and colleagues performed large-scale phage trials in a commercial shrimp hatchery using two lytic *V. harveyi*-specific broad host range bacteriophages. Phage application yielded 88% and 86% shrimp survival for each of the phages, while in antibiotic-treated (oxytetracycline and kanamycin) tanks, shrimp survival was 68% and 65%, respectively [79].

Phage therapy applications have shown promising results in other commercial species, such as sea cucumber, *Apostichopus japonicus* [87]. Three lytic bacteriophages (PVS-1, PVS-2 and PVS-3) were in all cases effective when tested in vitro against four pathogenic *V. splendidus* strains. Focusing on the preventive aspect of phage therapy, the authors prepared six different diets: a non-supplemented diet serving as control, an antibiotic-supplemented, three diets supplemented with single phages, and a diet that was supplemented with a cocktail of the three phages. Juvenile sea cucumbers were then fed on a daily basis. After 60 days, the animals were challenged by immersion in seawater containing *V. splendidus* for 2 days, and their survival rates were monitored for 10 days. The survival was 18% for the control diet, 82% for the antibiotics-supplemented diet, 65%, 58%, and 50% for the individual phage-supplemented diets and 82% for the phage cocktail-supplemented diet. In the same study, *V. splendidus* strain VS-ABTNL was injected in two groups of healthy sea cucumbers, while the control group was injected with sterile seawater. A phage cocktail was subsequently injected in one of the infected groups, and the survival rates were monitored for the following 10 days. All animals in the control group survived (100%), only 20% in the non-treated group and 80% survived in the phage treated group. Thus, it was concluded that phage cocktails could successfully protect *A. japonicus* against *V. splendidus* infection, and that both injection and immersion worked as delivery routes of phages. *V. splendidus* is a rapidly emerging pathogen, and the attempts for isolation of lytic phages have attracted a keen interest lately [92]. Similar phage trials in *A. japonicus* cultures have been performed against *V. alginolyticus* and *V. cyclitrophicus* using a mixture of two vibriophages and one vibriophage, respectively. In the former case, phage treatment at MOI = 10 led to 73% survival rates of the sea cucumbers, compared to only 3% survival that was observed in the non-treated group [86]. In the latter, the survival rate of juvenile *A. japonicus* was enhanced from 18% to 81% when fed with phage-containing feed, to 63% when injected with purified phage virions and to 58% when immersed in the phage-containing bath [88].

The bacteriophage CHOED has been tested for conferring protection against vibriosis in Atlantic salmon (*Salmo salar*) [89]. The presence of CHOED at MOI of 1 and 20 provided 100% protection of the fish against *V. anguillarum*, whereas untreated fish suffered over 90% mortality. When *S. salar* was challenged with *V. anguillarum* in aquaculture conditions, the administration of CHOED at MOI of 100 resulted in 100% fish survival 20 days after exposure to the pathogen, compared to only 60% survival in the non-treated fish.

The in vitro use of a phage cocktail with VP-1, VP-2, and VP-3 against *V. parahaemolyticus*, has been significantly more effective than using individual phages, albeit VP-3 was mainly responsible for the cocktail’s lytic activity [93]. Although the efficacy of the phages contained in the cocktail can vary, multivalent phage cocktails can be effective against several pathogenic strains of the host and they can greatly delay the development of resistance due to the different phage components. Moreover, the idea of a phage cocktail allows the use of lytic phages with narrow host ranges, since several of them can be combined to produce a much broader lytic spectrum [94].

Phage delivery methods are of vital importance for a successful therapy, and depend on the presence of the phages at the area of infection in a titer above the therapeutic threshold. Ryan and colleagues have reviewed the phage delivery routes in human phage therapy trials, and they concluded that parenteral injection is the most successful route of phage administration, because the phages can immediately reach the systemic circulation [95]. In several aquaculture phage therapy trials, administration of bacteriophages via injection has also been the most successful route of delivery, since bacteriophages could be detected in the fish tissues for several days after administration [76,96]. However, parenteral injection, apart from the fact that it is rather stressful for the animals, has significant limitations in its practical application when (1) fish or shellfish are too small or too numerous or (2) continuous treatment is required. In the majority of the in vivo trials, phages are added to the water simultaneously, or right after the bacteria. This method reduces the number of pathogens used for the challenge, which in turn results in lower infection rate. The oral route of delivery, the immersion in phage bath, and the addition of phages to the surrounding water are very common methods that often lead to high protection against bacterial pathogens [74,75,97] and greatly increase the applicability of phage therapy. Especially, administration of phages via phage-coated feed has been shown to be an efficient delivery method, resulting in constant, high abundance of phages in the fish organs for several weeks [97]. A variety, though, of delivery routes has been suggested in aquaculture phage trials, because bacterial infections can occur during all the developmental stages of cultured organisms; from the eggs to the broodstock [98].

**Figure 2:**
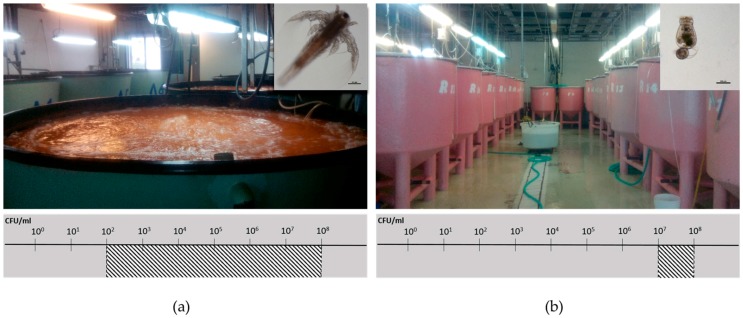
Facilities for live feed production from a commercial fish farm unit. (**a**) *Artemia salina* in culture tanks with vigorous aeration, where the native presumptive *Vibrio* load is regularly estimated between 10^7^ and 10^8^ cells per mL; (**b**) *Brachionus plicatilis* culture tanks, where the native presumptive *Vibrio* load is regularly between 10^2^ and 10^8^ cells per mL [103].

Reducing the number of vibrios in aquaculture environment is another strategy that has been examined. Pathogenic vibrios are present in live feeds offered to fish or invertebrate larvae, and the feed is thus a major source of pathogens entering the marine hatcheries [93,99]. Preventive administration of bacteriophages can be applied either directly to the environment of the cultured animal, preferably during early growth stages, or to the live prey, to control the source of pathogens to the hatchery facilities. *V. anguillarum*, *V. alginolyticus*, and *V. splendidus* are some common examples of pathogenic vibrios which are entering the aquaculture environment through live feeds, such as *Artemia salina* and *Brachionus plicatilis* (Figure 2) [52,100,101,102].

The *V. alginolyticus*-specific broad host range lytic phages *φ*St2 and *φ*Grn1 have been successfully used as a “smart” disinfectant that selectively reduces vibrios in live feeds. Since *V. alginolyticus* is a prevalent component in live feeds, such as *Artemia* and rotifers, a scheme of precautionary phage administration in *Artemia salina* live feed cultures was evaluated. A combination of these two phages was administered in *A. salina* live prey at MOI: 100, leading to a significant reduction of the native *Vibrio* load by 1.3 log units, suggesting a decrease in the risk of a vibriosis outbreak in the marine hatchery [104]. Further research on *φ*St2 and *φ*Grn1 has revealed that during infection, these phages are able to hijack and reprogram the host’s metabolic machinery, in order to meet their augmented demands for energy and nucleotide biosynthesis, making their therapeutic potential highly efficient [105].

### The Profile of a Good Candidate: KVP40 Case

Several issues are important when selecting safe and efficient candidates for phage therapy and next generation sequencing technology plays an important role in revealing the genomic composition and the lytic nature of viruses, which are main criteria in the selection process. The absence of genes related either to lysogeny or to any known toxins [106] confirmed the lytic nature of the vibriophage KVP40, making it a proper candidate for phage therapy trials against vibriosis. Phage KVP40 [106,107], is a myovirus classified in a group which has been designated as “schizoT4like” or “KVP40-like” [108]. It was isolated against a clinical *V. parahaemolyticus* strain, however, it has a broad host range, able to infect several other strains of eight different *Vibrio* species: *V. alginolyticus*, *V. cholerae*, *V. parahaemolyticus*, *V. anguillarum*, *V. splendidus*, *V. mimicus*, *V. natriegens*, and *V. fluvialis* [25,107]. The broad lytic spectrum and efficiency against several causative agents of vibriosis emphasizes the potential of KVP40 to control vibriosis in aquaculture settings.

The bacterial receptor that KVP40 recognizes in order to infect its hosts is the universal outer membrane protein K (OmpK), which is very common among *Vibrio* species [109]. Targeting a broadly distributed receptor is a key point when looking for a broad lytic spectrum bacteriophage; however, as it will be further discussed below, this can also result in the development of several defense strategies from the bacterial host in order to reduce the cost of resistance. In addition, KVP40 is a phage with a large genome (244,835 bp) able to take advantage of its host’s metabolic machinery in order to maximize the efficiency of the infection, and thus, the overall impact of phage therapy. Lytic bacteriophages can manipulate and reprogram the host’s metabolic machinery in order to support and facilitate their own DNA replication and protein synthesis, which are necessary for the packaging and release of the new virions [110,111]. They can mediate a transition from a host-oriented to a phage-oriented metabolism [112,113] during infection, since the interactions of their early phage genes with DNA metabolism-involved host proteins, cease the host replication [110]. KVP40 was found to encode a functional NAD^+^ salvage pathway, which can boost its own replication during infection. This pathway is also conserved in other large genome phages that carry similar genes involved in nucleotide metabolism [114]. Last but not least, many phages, including KVP40, carry a high number of tRNAs, which may provide the phage with a small degree of autonomy when it comes to the translation of its own genes [115].

## 4. Issues Raised in Phage Therapy

As evident from above, phage therapy is definitely an attractive alternative to combat pathogenic bacteria, which may be used not only as a treatment, but also to prevent infections. However, there are several important constraints, such as the phage efficacy under aquaculture conditions, administration methods and persistence of phages in the system, the possibility of unwanted phage-encoded properties and, perhaps most importantly, the development of phage resistance, that need to be evaluated before a phage therapy application scheme can be considered successful.

### 4.1. Phage Therapy from the Lab to the Field

During the stages of a therapeutic phage suspension development in the lab, host specificity, life cycle parameters and lytic nature of the phage, are the main prerequisites that need to be covered. The selection of appropriate phages that are going to be used alone or forming a phage cocktail is also crucial for the outcome of the phage therapy. However, despite the promising results that some lytic bacteriophages have shown under laboratory conditions, application of phage-based treatment in aquaculture settings is associated with a number of additional challenges that need to be addressed.

Previously, reporting of phages with low in vivo activity has been one reason for questioning globally the actual efficacy of phage therapy against bacterial infections in animals and humans [116,117]. The optimal phage delivery method (injection, oral, immersion) may vary between different aquaculture settings, and should be carefully determined in each case. For instance, although injection has been mentioned as the most effective delivery route [95], immersion of the cultured animals in phage-containing water has been also quite effective, since bacteria begin their infection cycle from adhering to the mucosa of the fish, which constitutes the first physical and chemical barrier of fish against pathogens [118]. Marine fish species drink water to maintain their internal ionic balance, and therefore, phages of the water will have the opportunity to encounter pathogenic bacteria for which the infection route is through the fish intestinal mucosa. Even when bacteria attack the intestinal mucosa, fish drink a lot of water, so phages still encounter intestinal bacteria [97]. In vitro results based on immersion are very often similar to those obtained in vivo, since this approach, in both cases, is based on phage-bacteria interactions that take place in a phage-containing suspension [119]. Quantification of the viruses, in the animal tissues or in the aquatic environment where therapy was applied, will define the efficacy of the delivery route. However, repeated phage administration using either delivery route has been the most effective way to maintain a high bacteriophage titer in the system [77,87,97]. Oral administration via phage-coated fish pellets is a quite feasible and effective way to keep a constant phage input to the system with minimum effort, and easily incorporated into the daily routine of the fish farm [97]. Furthermore, as the pathogens may be present in different stages of the production process, it is important to consider where in the production the addition of phages is expected to most efficiently reduce the pathogen (i.e., disinfection of live feed, disinfection of fish eggs, treatment of infected fish, etc.).

### 4.2. Concerns about Phage-Treated Organisms

A bacterial lysate might contain endotoxins which, if not removed, may be fatal for the cultured organism [120,121]. The phage stocks that are administered to the cultured organisms should therefore be meticulously prepared to remove bacterial debris, secondary metabolites, enzymes, etc., that might potentially be toxic for the fish or shellfish [122]. Endotoxin-free phage suspensions are regularly produced today [123,124], eliminating potential side effects that may create unnecessary consideration to legislation and public opinion about phage therapy. Another concern about phage therapy in organisms such as fish, which have an adaptive immune system, is the potential immunological response of the phage-treated organism, that might trigger the production of phage-neutralizing antibodies, decreasing in vivo phage efficacy [125,126]. This possibility in aquaculture has been examined after phage-coated feed administered in yellowtail, *Seriola lalandi* [72] and intramuscular phage injection in ayu, *Plecoglossus altivelis* [127], however, phage-neutralizing antibodies were not detected in the studies. Production of such antibodies after phage administration in aquaculture is not yet documented in the literature [74].

### 4.3. Development of Resistance

**Figure 3:**
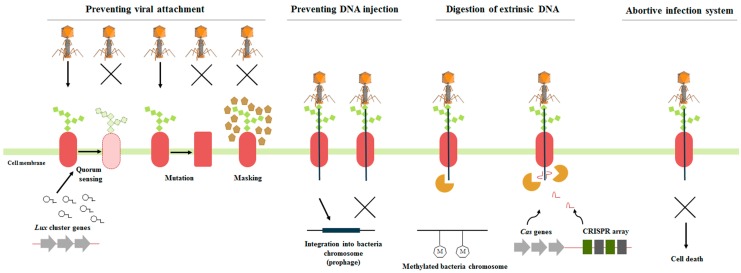
Overview of the main phage defense mechanisms in bacteria. Prevention of viral attachment on the bacterial surface can be achieved by mutating or masking the receptors, as well as downregulation of receptor expression, orchestrated by quorum sensing (QS). DNA injection may be successfully averted by superinfection exclusion (Sie) mechanisms. If phage DNA enters the bacterial host, its digestion can be catalyzed by R-M mechanism and CRISPR-Cas arrays systems. Deliberate death of the infected cell (abortive infection) constitutes another strategy against viral predators, where prevention of phage proliferation reduces spreading of the infection to the rest of the population.

Development of resistance is probably the most significant limitation in the whole concept of phage therapy. In the ocean, phages and their bacterial hosts are in a perpetual arms race, under strong evolutionary pressure [128,129]. Although the use of phage cocktails can reduce or delay the emergence of resistant strains [93,130], bacteria have developed several strategies (Figure 3) to cope with their viral predators [131,132,133].

#### 4.3.1. Preventing Viral Attachment 

The most crucial step for the successful infection of a bacterial host by a phage is its adsorption by the host through a specific reaction between the phage receptor-binding protein and the bacterial cell surface receptor. There is a great variety of components on the bacterial surfaces that are targets for phage attachment, such as proteins, polysaccharides, and lipopolysaccharides [134]. However, bacteria have developed strategies to effectively prevent phage adsorption events by (a) modifying the phage receptors by mutational changes, (b) masking receptors by producing an extracellular matrix, (c) producing competitive inhibitors, or (d) losing or downregulating the expression of the receptor [131,133]. In several cases the regulation of surface modifications is orchestrated by QS [135]. For instance, in the case of KVP40 phage infecting *V. anguillarum*, QS is used by bacteria in order to select between two different protection mechanisms according to their population density. At low host densities, the OmpK receptor for KVP40 is fully expressed, and the bacteria are protected from infection by forming aggregates and biofilms. At high host densities, the OmpK receptor is downregulated through a QS regulation pathway, making *V. anguillarum* less susceptible to the phage [24]. In *V. cholerae*, a different mechanism has been described, where surface modification of the phage receptor prevents attachment of the lytic phage ICP2 [136]. However, bacteriophages can regain their ability to attach on their targets by modifying their receptor-binding proteins and getting access to the modified bacterial cell surface receptors [137].

#### 4.3.2. Preventing DNA injection

Even if the adsorption of the phage has been successful, bacteria have developed strategies to prevent the injection of the incoming viral DNA. Superinfection exclusion (Sie) systems are based on proteins related to cell surface modifications or inhibition of replication, and are often encoded by prophages or plasmids. Sie systems can provide immunity to the prophage-carrying bacterium against a second potential infection by similar bacteriophages [133,138,139]. A subcategory of Sie is the repressor-mediated immunity, where the repressor protein retains the prophage in the lysogenic cycle while providing immunity against an infective phage carrying the same type of repressor [140]. In a recent study, several temperate *V. anguillarum*-specific bacteriophages, designated as H20-like phages, were shown to contain a lambda-like cI repressor gene in their genomes. It was suggested that this mechanism possibly confers repressor-mediated immunity to other H20-like phages in their *V. anguillarum* host [141]. A Sie system-encoding prophage has recently been reported to confer phage resistance in the *V. cholerae* strain 919TP [142]. Compared to receptor alteration strategies, which prevent phage adsorption, and thus only protect the individual resistant cell, the Sie systems immobilize and inactivate the phage, thereby reducing the infection load on the remaining population [143].

#### 4.3.3. Digesting Extrinsic DNA

In cases where the phage DNA enters the bacterial host, bacteria have developed several mechanisms for its inactivation: (a) Restriction-modification (R-M) systems and (b) CRISPR-Cas system [131]. R-M systems (type I, II, III, and IV) are composed by a methyltransferase and a restriction endonuclease, which catalyze the methylation of the bacterial DNA and the cleavage of the viral, unmethylated DNA, respectively [144,145,146]. Almost all bacterial genera carry R-M systems [133], and it is hypothesized that high levels of horizontal gene transfer (HGT) are responsible for their spreading and evolution among prokaryotes [145]. Vibriophage KVP40 has been reported to be restricted and modified by the R-M system of at least five *Vibrio* species [147]. However, phages have simultaneously evolved to evade the omnipresent R-M systems. Approximately, 20% of the available phage genomes carry methyltransferase-encoding genes, suggesting the ability to protect their own genome through methylation [141,148]. Methyltransferases can also affect bacterial virulence [149] or they may function as transcriptional regulators by either activating or repressing bacterial genes [150,151]. N6-adenine methyltransferase was previously found in the temperate vibriophage VHML, where it was linked to the virulence of *V. harveyi* host strain upon integration [152]. H20-like vibriophages were also found to carry a N6-adenine methyltransferase gene; however, its exact function still needs to be explored [141]. Phages may methylate parts of their genome, preventing its degradation by the host’s restriction enzymes, whereas methylation could modify the properties of the host. It has been reported that viral infection of specific bacterial hosts subsequently affected the host range of the newly produced virions [153,154], suggesting that specific differences in the methylation of viral DNA during phage production affects the infectivity of the produced phages. Integrating conjugative elements (ICEs), ICE*Vsp*Por3 and ICE*Val*Spa1, that were recently identified in *V. splendidus* and *V. alginolyticus*, respectively, harbor genes encoding distinct R-M systems, which are able to confer protection against viral infection when expressed in *Escherichia coli* [155].

CRISPR-Cas has been described as an adaptive immune system of bacteria. This system is composed of (1) *cas* genes, which are responsible for the expression of the protein machinery that performs the immune response, and (2) the CRISPR loci, composed of 21–48 bp direct repeats interspaced by non-repetitive spacers of 26–72 bp, which provide genetic memory of previous viral infections. Although there are two classes, six types, and 16 subtypes of CRISPR-Cas systems [156], all of them are based on three common functional phases: (1) adaptation-spacer(s) from invasive DNA are acquired and then integrated into the CRISPR loci of the bacterial genome, (2) expression—transcription of the CRISPR loci that encode a CRISPR RNA (crRNA) molecule which will be combined with Cas protein forming crRNA–Cas complexes, and (3) interference—the crRNA-Cas complexes attach and digest complementary nucleic acids, providing immunity to the bacterial host [131]. Among the sequenced bacterial genomes, CRISPR loci are found in approximately 40% of them [157]. Although CRISPR-Cas systems are highly sophisticated, their conferred immunity can be bypassed by the bacteriophages by mutations or deletions in the targeted proto-spacer in the phage genome. Single nucleotide mutations in the protospacer genomic region of *S. thermophilus* phages were able to circumvent their bacterial host’s CRISPR-Cas defense system [158,159]. Screening of the 1935 publicly available *Vibrio* genomes using CRISPRfinder [160] showed that CRISPR(s) were present in 278 (14.4%) genomes. Most of the CRISPR-containing *Vibrio* strains had one CRISPR array, but some carried up to 11 [161]. In total, 388 CRISPR arrays were identified in all the *Vibrio* genomes The CRISPR prevalence in the *Vibrio* genus is thus substantially lower than the reported 50% in bacteria in general [156]. Within the 28 genome-sequenced *V. anguillarum* strains, only one strain, *V. anguillarum* PF7, contained CRISPR. Out of the two CRISPR-Cas arrays of *V. anguillarum* PF7, eighteen spacers were >95% similar to genomic parts of the H20-like vibriophages, with eight of them being 100% identical [141]. The knowledge of the contribution of CRISPR-Cas systems in vibrios’ defense is sporadic, and needs to be more thoroughly evaluated. It is also worth mentioning that the *V. cholerae* phage ICP1 is the first bacteriophage recorded to encode its own CRISPR-Cas arrays as a counter-defense against *V. cholerae* phage inhibitory chromosomal island [162].

#### 4.3.4. Abortive Infection System

Bacterial infection by phages can sometimes be non-productive, even though it leads to the death of bacterial host. This kind of abortive infection may be the result of “lysis from without” (LO), where extensive simultaneous infections (i.e., very high MOI) may destroy the cell membrane. In another type of abortive infection leading to non-productive phage infections, the infected bacteria can enter a programmed cell death, and thereby prevent the spreading of the infection to the neighboring cells. This altruistic behavior is orchestrated by the abortive infection (Abi) system [131,163]. Although the best characterized Abi system is the Rex system, which is found in phage λ-lysogenized *E. coli* preventing infection by other coliphages [164,165], and most Abis have been identified in plasmids of Gram-positive strains [163], *V. cholerae* has also been documented as a carrier of such a system [166]. However, it has been proven that some Abis, such as TA system ToxIN described in *Pectobacterium atrosepticum*, may act also through toxin-antitoxin (TA) mechanisms, aborting phage infection [167]. TAs were initially found in plasmids, but are now known to be abundant in bacterial genomes. The components of these systems, a toxin and an antitoxin, neutralize each other, keeping the balance that maintains bacterial life. Upon phage infection, toxin and antitoxin production stops, yet antitoxin degrades faster, allowing the toxin to kill the bacterial cell [168]. For instance, MosAT TA system in *V. cholerae* resembles AbiE, a broadly distributed TA-Abi system in bacterial genomes inducing bacteriostasis and conferring phage resistance [168]. A high number of both confirmed and putative TA systems have been reported in *V. cholerae* and *V. parahaemolyticus* [169,170], however, their potential functionality as Abi systems still needs to be elucidated.

Even though the result of abortive infections is lysis of the bacterial cell, the phenomenon limits self-adjusting properties of the phages because there is no virion production. The implication of this defense mechanism in vibrios needs to be evaluated in vitro, during the assessment of the viral lytic spectrum.

#### 4.3.5. Resistance Comes at a Cost

Thinking of the variety of phage defense mechanisms identified in bacteria, one might wonder what determines their distribution among bacteria, and why not all bacteria carry all of them. The explanation is that development of resistance comes at a fitness cost for the bacteria [171], so they need to be parsimonious when they invest on resistance strategies. Phage-binding bacterial receptors, which serve as recognition points for the phages, are often also associated with important cell functions, such as nutrient uptake, motility, and ability to attach on surfaces [172,173,174]. Therefore, surface modifications may significantly reduce bacterial fitness, including a reduced colonization ability [175,176] and increased susceptibility to other phages [177]. Loss of virulence and gliding motility has been observed in phage resistant *Flavobacterium columnare* [178], and similar results were recorded in phage-resistant *Flavobacterium psychrophilum* strains, which have shown decreased hemolytic activity, gelatinase activity, and total protease activity, as well as mutations in significant virulence genes [179]. Accordingly, in vibrios, phage-resistant strains have been shown to be less harmful against their eukaryotic host. In experimentally challenged pipefish (*Syngnathus typhle*), three *Vibrio* spp. Isolates, representing different phage susceptibility, showed positive correlation between phage susceptibility and virulence [180]. Further, loss of virulence was observed in four KVP40-resistant *V. anguillarum* strains as demonstrated by reduced mortality of cod (*Gadus morhua*) larvae challenged with phage-resistant clones, compared to a control group challenged with the wild type *V. anguillarum* strain [181].

Bacteria and phages are in a perpetual arms race, so phages also evolve counter-defensive strategies to circumvent bacterial defense mechanisms. The fitness cost, including loss of virulence, which is often associated with resistance, constitutes a barrier to the prevalence of these defense mechanisms [131], and selection pressure on different anti-phage strategies depends on the trade-off between mortality imposed by phages and fitness cost of the defense strategy, under the given environmental conditions. Hence, constitutive defense strategies, such as mutating the bacterial receptors and inducible defense strategies, such as CRISPR-Cas systems, may prevail under different conditions and for different phage-host interactions. In the former case, modification of the receptors is permanently associated with a substantial fixed cost that directly affects bacterial fitness, while in the latter case, bacterial CRISPR-Cas systems may be elicited only upon viral infection [182]. The force of infection and the nutrient availability are usually the most important factors that will determine the bacterial decision between constitutive and inducible defense mechanisms [182,183,184]. The CRISPR-Cas system is favored when the frequency of infection is low and the nutrients are limited, because it is associated with a lower cost for the bacterial cell. Viruses can, though, quickly mutate and escape, suggesting that these systems would be most effective when exposed to low phage diversity, due to relatively limited capacity for spacer acquisition. Accordingly, genomic mapping of CRISPR spacers and viral genomes has shown that only recently acquired resistance was functional for phage defense [185]. On the other hand, surface modifications are favored when frequency of infection is high and nutrients are abundant, because bacteria need to be in an “always on” defensive position, even if the costs are high [182].

Acquiring resistance against more than one bacteriophage might increase the fitness cost for the host, since it will need to modify different phage-binding surface receptors [175,186]. Therefore, coping with phage cocktails implies a higher fitness cost for the bacteria. Furthermore, the accumulating indications that phage-resistance leads to dominance of less virulent phenotypes, suggest that the problems with resistance associated with phage treatment in aquaculture may be limited. Indeed, the fitness cost of phage immunity needs to be comprehensively explored, with special emphasis on the virulence properties of phage resistant pathogens, potentially allowing the prediction of the implications of the development of presumably less virulent phage resistant bacteria for the survival of phage treated animals.

## 5. Temperate Vibriophages and Lysogenic Conversion

Another concern when selecting phages for therapeutic purposes is the risk of using phages encoding unwanted genes that may spread in the pathogen population. Consequently, it must be explicit that only lytic bacteriophages should be considered as potential candidates.

However, temperate bacteriophages are also a vital part of the natural virome, and key players in driving bacterial evolution by disseminating genetic information through horizontal gene transfer (HGT). HGT may take place in both lytic and temperate phage with the form of generalized transduction, whereas the events of specialized transduction and lysogenic conversion are restricted only to temperate phages [187,188]. The high rate of generalized transduction events, which also applies to lytic phages, has lately raised significant concerns against phage therapy, since virulence or resistance genetic element might spread among the pathogenic bacteria [189]. Complementary researches on *E. coli* and *Streptococcus pyogenes*, though, have shown that antibiotic resistance genes may be disseminated only through temperate transducing bacteriophages [190,191,192]. However, it was recently found that two lytic bacteriophages against *E. coli*, designated as “superspreaders”, could promote extensive plasmid transformation, and therefore efficiently disperse antibiotic resistance genes [193]. Hence, it is crucial that such phages are avoided in phage therapy or other medical applications.

A serious constraint in phage therapy is, therefore, the unsafe use of temperate phages as therapeutic agents. The process through which prophages integrate in their host’s genome and transfer genes whose expression may render increased bacterial fitness, either directly (e.g., phage-encoded toxins) or indirectly (e.g., increased fitness during infection) is designated as lysogenic conversion. This process possesses a dominant role in conferring enhanced fitness to the prophage-carrying bacteria. The importance of this process is highlighted in vibrios, since there is a plethora of vibriophages where lysogenic conversion has positively affected, both directly and indirectly, the fitness of their lysogenized hosts. A classic example of lysogenic conversion in vibrios is the phage-mediated production of cholera toxin, by the filamentous phage CTXΦ [194]. However, there are several examples of lysogenic conversion in other *Vibrio* pathogens. *V. harveyi* strains carrying the prophage VHML, were able to metabolize fewer nutrient sources than their uninfected counterparts [195]. Switching off unnecessary bacterial functions would make lysogenized *V. harveyi* strains less energy-consuming, hence more competitive under nutrient-limited environments [196]. The same temperate phage VHML was reported as being responsible for conferring virulence to the *V. harveyi* strain 642, since avirulent *V. harveyi* strains were converted to virulent, when infected by phage VHML [78,197]. A similar observation was made in the case of the prophage VOB, which was integrated in the genome of its *V. owensii* host. After the induction of VOB in the lab, it was co-cultured with naïve *V. harveyi* and *V. campbellii*. The avirulent vibrios were lysogenized by the induced VOB and they became virulent, causing increased mortality to *Penaeus monodon*. It was concluded that VOB was responsible for some of the virulence of *V. owensii*, as well as for the acquired virulence of *V. harveyi* and *V. campbellii* lysogens [198]. In a very recent study, prophage-like elements that were identified in the genomes of *V. anguillarum* strains T265 and Ba35, contained genes related to zonula occludens toxin (Zot), implying the contribution of the prophage to bacterial virulence [199]. Prophages K139 and VIPΦ have also been reported to increase the pathogenesis of their *V. cholerae* hosts by increasing both virulence and colonization ability [200,201]. Lysogenic conversion may be implicated also in bacterial host’s defense against viral infection. Phage-induced lysis of some cells could release prophage-encoded toxins, such as colicins, that might eliminate competitors, helping the rest of the bacterial population to take advantage of the environmental niche and resources [202,203]. In a recently published study, the tail length tape measure gene that was identified in the genome of the H20-like temperate vibriophages resembled the structure of channel forming toxin colicin Ia, hence, an additional role of the gene in bacterial competition was suggested by the authors [141]. Furthermore, Sie and Abi defense systems that were previously mentioned, are also among the beneficial effects that prophages impart to their hosts [204].

Although temperate bacteriophages are not suitable for therapeutic purposes, lysogenic lifestyle is the symbiotic aspect of the virus-bacteria interactions which has been the outcome of their refined co-evolutionary relationships [205]. Already in 1961, Campbell suggested a beneficiary contribution of prophages to their host by stating “One therefore must look for possible means by which the phage might impart a selective advantage to its host” [206].

## 6. Perspectives on Phage Therapy Today

Wherever bacteria thrive, so do predatory phages. During 2017, we celebrated 100 years from the discovery of the bacteriophages and the idea of using specific bacteriophages as a weapon to biologically control pathogenic bacteria. Phage therapy approaches against bacterial infections have been revived, primarily due to major problems with antibiotic-resistant bacteria we are facing as a result of excessive usage of antibiotics. In addition, the increasing temperature in the oceans, the fatal effects of vibriosis on the global aquaculture industry, as well as a plethora of different vibrios that may trigger the disease has further emphasized the need for exploring the potential of phages to control vibrio pathogens.

While the initial idea behind phage therapy was treatment of diseases, as in the case of antibiotics, future work should include prophylactic use of lytic bacteriophages to reduce the pathogen load and reduce the risk of infection. In marine aquaculture, addition of bacteriophages to live feeds, such as *Artemia* and rotifers, may be an efficient way to selectively disinfect the life feeds immediately prior to entering fish or invertebrate production cycle. Since both *Artemia* and rotifers are produced in batch cultures with a short retention time, the risk of resistance development is minimal, as opposed to providing phages prophylactically in the feeds of fish, or directly in rearing tanks, which would allow for phage-bacteria co-evolution. However, application of lytic phages as fish feed additives may be an efficient way to prevent the pathogens from establishing in the fish organs. Recirculating aquaculture systems (RAS) could be ideal environments for the application of phage therapy, since the water exchange is quite limited. Additionally, the combinatory usage of bacteriophages, together with another ecologically friendly alternative such as probiotic bacteria, constitutes a strategy that would be expected to be highly effective against bacterial diseases. Combining biological approaches with different targets and modes of action may minimize the risk of future resistance development, as has been seen in human medicine, where combined drugs are successful in antibacterial and antiviral treatment.

More research is still required to optimize the phage application under field conditions (phage composition, timing of application, delivery etc.) and to eliminate the potential risk factors associated with phage application (dispersal of unwanted genes, effects on fish microbiota). Further investigation of the naturally occurring phages in the cultured animals’ microbiota is going to unravel their role in the organism’s protection against bacterial diseases, and evaluate the possibility of them being used in a more targeted phage therapy scheme. This requires extensive sequencing of viral genomes and analyses for presence of genetic elements that might potentially interfere with the bacterial fitness or affect the organism’s health. Such knowledge would also provide us a window for visualizing a future of molecularly engineered lytic virions. Consequently, more in vitro and in vivo test trials are required before the final release, and any side effects need to be meticulously recorded. However, facing a future with increasing problems with antibiotic resistant pathogens, exploring phage-based alternatives is now more necessary than ever.

## Figures and Tables

**Table 1:** Phage therapy trials against causative agents of vibriosis in experimental aquaculture setups.

Cultured Animal	Causative Agent	Reference
*Penaeus monodon*	*V. harveyi*	[77,78,79,80,81]
*Haliotis laevigata*		[82]
*Panulirus ornatus*		[83]
*Ostrea plicaltula*	*V. parahaemolyticus*	[84]
*Litopenaeus vannamei*		[85]
*Apostichopus japonicus*	*V. alginolyticus*	[86]
*Apostichopus japonicus*	*V. splendidus*	[87]
*Apostichopus japonicas*	*V. cyclitrophicus*	[88]
*Salmo salar*	*V. anguillarum*	[89]
*Danio rerio*		[90]
*Acropora millepora*	*V. coralliilyticus*	[91]
